# P188: Assessment model of hospital infection control programs: validation of measurement properties

**DOI:** 10.1186/2047-2994-2-S1-P188

**Published:** 2013-06-20

**Authors:** RA Lacerda, CPR Silva

**Affiliations:** 1 Medical-Surgical Department, Nursing School of University of São Paulo, São Paulo, Brazil

## Introduction

The study aimed a system for assessing Hospital Infection Control Programs-HICPP, which enable application in situational diagnosis, whose results provide both improvements in the area and reliable information about the quality of these HICPPs in healthcare facilities.

## Objectives

Fully validate and test the reliability of measurement properties.

## Methods

Methodological development study by the construction and validated four indicators: PCET-Technical-operational structure of the HICPP; PCDO-Operating Guidelines for Control and Prevention of HI; PCVE-Epidemiological Surveillance System; PCCP-Prevention and Control Activities. The indicators were applied in 50 healthcare facilities of São Paulo-Brazil(2011). Internal consistency was analyzed by Cronbach a coeficient; the discriminant validity was carried out by comparing the scores of the indicators between two groups of hospitals (those which had some type of accredtiation versus those which did not) and exploratory factor analysis with tetrachoric correlation matrix was used to analyze the validity of the construct.

## Results

The indicators PCET and PCVE varied little, with almost 100% conformity throughout the sample, whereas the PCDO and PCCP presented good internal consistency with a variation of 0.67 to 0.80; discriminant validity showed higher average scores of conformity and were statistically significant in the group of institutions with accreditation; in the validation of the construct it was possible to differentiate 2 dimensions for PCDO (1-recommendations for prevention of HI and 2- recommendations for the standardization of prophylaxis procedures), with good correlation of the units of analysis that composed it. The same occurred for PCCP (1-interface with treatment units and 2-interface with support units). All of the indicators, except the PCCP, which ranged from 9.5% to 100%, presented scores of > 90%, which show that the HICPPs of participating hospitals have a good standard of quality, with higher average scores in the institutions with accreditation.

## Conclusion

The study enabled the validation of the measurement properties of the HICPP indicators and produced a HICPP assessment tool in an ethical and scientific manner for diagnosis of quality in this area.

## Disclosure of interest

None declared

